# Supplementary figures and images for: High-speed trains versus air transport vectors for mass transfers of critically ill patients: The TRANSCOV cohort study
Source: PLoS One. 2026 Apr 28;21(4):e0348090. doi: 10.1371/journal.pone.0348090 (PMC13123964; doi:10.1371/journal.pone.0348090)

**S1 Fig. Routes taken by patients involved in the study. Train group n=130. Air group n=163.**

**B**

**A**


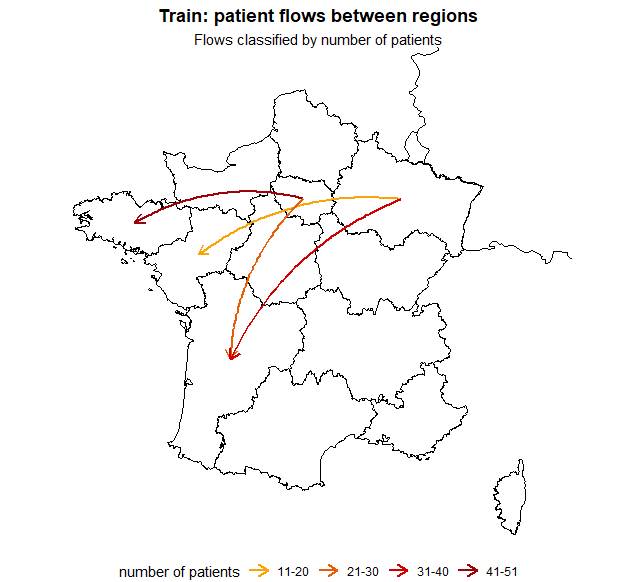

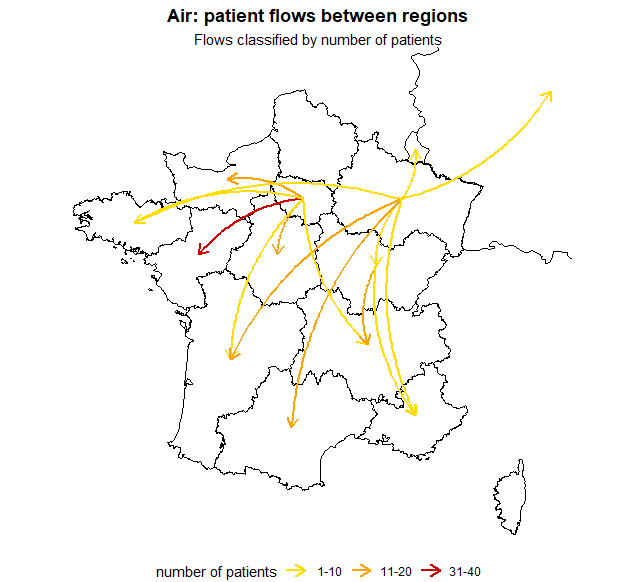

Supplement: S1 Fig — (DOCX) [file pone.0348090.s001.docx]
