# Supplementary material for: High-speed trains versus air transport vectors for mass transfers of critically ill patients: The TRANSCOV cohort study
Source: PLoS One. 2026 Apr 28;21(4):e0348090. doi: 10.1371/journal.pone.0348090 (PMC13123964; doi:10.1371/journal.pone.0348090)
Supplement: S2 Table — (DOCX) [file pone.0348090.s002.docx]

**S2 Table. Demographic and clinical profiles at the origin ICU and transfer characteristics of included and excluded patients.**

| Variables | Included patients  N = 293 | Excluded patients  N = 209 | *P*-value^a^ |
| --- | --- | --- | --- |
| Patient characteristics |  |  |  |
| Age (years) | 63 (53–69) | 63 (56–69) | 0.34 |
| Men | 213/293 (72.7) | 162/209 (77.5) | 0.22 |
| Weight (kg) | 81 (73–90) [17] | 85 (75–98) [13] | 0.004 |
| Risk factors |  |  |  |
| Current smoking | 12/275 (4.4) | 8/186 (4.3) | 0.97 |
| Chronic alcoholism | 12/271 (4.4) | 1/191 (0.5) | 0.01 |
| Comorbidities |  |  |  |
| Any comorbidity | 199/293 (67.9) | 128/206 (62.1) | 0.18 |
| No. of comorbidities | 1 (0–2) [9] | 1 (0–2) [7] | 0.07 |
| Diabetes | 90/292 (30.8) | 53/205 (25.9) | 0.23 |
| Hypertension | 151/292 (51.7) | 100/208 (48.1) | 0.42 |
| Cardiovascular disease^b^ | 34/288 (11.8) | 17/207 (8.2) | 0.19 |
| Asthma or COPD | 30/288 (10.4) | 19/208 (9.1) | 0.64 |
| Functioning limitation (KNAUS score) | 104/267 (39.0) | 76/195 (39.0) | >0.99 |
| Clinical status at admission in the origin ICU |  |  |  |
| Disease duration before ICU admission (days) | 9.0 (6.0–12.0) [10] | 9.0 (6.0–11.0) [13] | 0.94 |
| SAPS II | 40.0 (32.0–50.0) [35] | 37.0 (31.0–46.0) [38] | 0.12 |
| Intubated at admission | 134/292 (45.9) | 101/207 (48.8) | 0.52 |
| Transfer characteristics |  |  |  |
| Period of transfer |  |  | <0.0001 |
| 13–26 March 2020 | 44/293 (15.0) | 54/209 (25.8) |  |
| 27 March to 2 April 2020 | 149/293 (50.9) | 130/209 (62.2) |  |
| 3–10 April 2020 | 100/293 (34.1) | 25/209 (12.0) |  |
| Origin region |  |  | <0.0001 |
| Bourgogne Franche Comté | 27/293 (9.2) | 18/209 (8.6) |  |
| Corse | 0/293 (0.0) | 10/209 (4.8) |  |
| Grand Est | 103/293 (35.2) | 151/209 (72.2) |  |
| Ile de France | 163/293 (55.6) | 30/209 (14.4) |  |
| Destination region |  |  | <0.0001 |
| Abroad | 9/293 (3.1) | 132/196 (67.3) |  |
| Northwest region | 129/293 (44.0) | 20/196 (10.2) |  |
| Southwest region | 94/293 (32.1) | 24/196 (12.2) |  |
| Southeast and central regions | 61/293 (20.8) | 20/196 (10.2) |  |

Data are reported as median (IQR) [n missing] or n/total n (%). COPD, chronic obstructive pulmonary disease; CT, computed tomography; ICU, intensive care unit; IQR, interquartile range; SAPS, Simplified Acute Physiology Score.

**^a^***P*-values were calculated with chi-square test or Fisher exact test for discrete variables and with Wilcoxon-Mann-Whitney test for continuous variables.

^b^Cardiovascular disease included either ischaemic heart disease, heart failure or stroke.
