# Supplementary material for: High-speed trains versus air transport vectors for mass transfers of critically ill patients: The TRANSCOV cohort study
Source: PLoS One. 2026 Apr 28;21(4):e0348090. doi: 10.1371/journal.pone.0348090 (PMC13123964; doi:10.1371/journal.pone.0348090)
Supplement: S3 Table — (DOCX) [file pone.0348090.s003.docx]

**S3 Table. Comparison of patient profiles at the origin ICU according to recorded or inferred transport vectors.**

| Variables | Recorded group  N = 193 | Inferred group  N = 100 | *P*-value ^a^ |
| --- | --- | --- | --- |
| Patient characteristics |  |  |  |
| Age (years) | 65 (57–71) | 62 (55–71) | 0.14 |
| Men | 141/193 (73.1) | 72/100 (72.0) | 0.85 |
| Weight (kg) | 80 (72–90) [14] | 82 (75–90) [3] | 0.26 |
| Risk factors |  |  |  |
| Current smoking | 5/183 (2.7) | 7/92 (7.6) | 0.11 |
| Chronic alcoholism | 9/182 (4.9) | 3/89 (3.4) | 0.76 |
| Comorbidities |  |  |  |
| Any comorbidity | 132/193 (68.4) | 67/100 (67.0) | 0.81 |
| No. of comorbidities | 1 (0–2) [6] | 1 (0–2) [3] | 0.80 |
| Diabetes | 61/192 (31.8) | 29/100 (29.0) | 0.63 |
| Hypertension | 97/193 (50.3) | 54/99 (54.5) | 0.49 |
| Cardiovascular disease^b^ | 25/190 (13.2) | 9/98 (9.2) | 0.32 |
| Asthma or COPD | 20/191 (10.5) | 10/97 (10.3) | 0.97 |
| Functioning limitation (KNAUS score) | 66/177 (37.3) | 38/90 (42.2) | 0.43 |
| Clinical status at admission in the origin ICU |  |  |  |
| Disease duration before ICU admission (days) | 8.0 (6.0–12.0) [5] | 9.0 (6.0–11.0) [5] | 0.87 |
| SAPS II | 40.0 (32.0–49.0) [30] | 40.0 (32.0–52.0) [5] | 0.87 |
| Intubated at admission | 87/193 (45.1) | 47/99 (47.5) | 0.70 |

Data are reported as median (IQR) [n missing] or n/total n (%). COPD, chronic obstructive pulmonary disease; CT, computed tomography; ICU, intensive care unit; IQR, interquartile range; SAPS, Simplified Acute Physiology Score.

**^a^***P*-values were calculated with Chi-square test or Fisher's exact test for discrete variables and with Wilcoxon-Mann-Whitney test for continuous variables.

^b^Cardiovascular disease included either ischaemic heart disease, heart failure or stroke.
