# Supplementary material for: High-speed trains versus air transport vectors for mass transfers of critically ill patients: The TRANSCOV cohort study
Source: PLoS One. 2026 Apr 28;21(4):e0348090. doi: 10.1371/journal.pone.0348090 (PMC13123964; doi:10.1371/journal.pone.0348090)
Supplement: S4 Table — (DOCX) [file pone.0348090.s004.docx]

**S4 Table. Association between co-variables included in the fully adjusted model and length of stay at the destination ICU.**

|  | Crude association | | | Adjusted association  N = 257^a^ | |
| --- | --- | --- | --- | --- | --- |
| Variables | N | Exp(beta)  [95% CI] | *P*-value | Exp(beta)  [95% CI] | *P*-value |
| Clinical events |  |  |  |  |  |
| Acute kidney injury | 291 | 1.57 [1.31–1.88] | <0.0001 | NA | NA |
| Nosocomial infection | 291 | 1.98 [1.7–2.29] | <0.0001 | 1.21 [1.08–1.35] | 0.001 |
| Shock | 290 | 1.67 [1.38–2.02] | <0.0001 | NA | NA |
| Thromboembolic event | 289 | 1.56 [1.29–1.89] | <0.0001 | 1.15 [1.02–1.30] | 0.028 |
| Delirium | 289 | 1.20 [1.00–1.45] | 0.06 | 1.20 [1.07–1.35] | 0.002 |
| Neuromyopathy | 288 | 1.78 [1.52–2.10] | <0.0001 | NA | NA |
| Treatments |  |  |  |  |  |
| Neuromuscular blocking agents | 289 | 1.66 [1.38–1.98] | <0.0001 | 1.14 [1.02–1.28] | 0.02 |
| Prone position | 291 | 1.76 [1.51–2.05] | <0.0001 | NA | NA |
| Tracheotomy | 291 | 2.11 [1.75–2.55] | <0.0001 | 1.30 [1.12–1.51] | 0.0006 |
| Mechanical ventilation duration >10 days  vs ≤10 days | 263 | 2.82 [2.53–3.14] | <0.0001 | 2.33 [2.08–2.60] | <0.0001 |

Results were from linear regression models using log-transformed length of stay as dependent variable. Therefore, exponentiated beta can be interpreted as a multiplicative factor. For example, length of stay was 21% longer [(1.21 -1.00) x 100] for patients with nosocomial infection. Exp(beta): exponentiated beta.

^a^Adjusted for vector, patient characteristics and transfer characteristics with backward stepwise elimination of variables. The final adjusted model included exposure (air/train), initial intubation, nosocomial infection, thromboembolic event, delirium, neuromuscular blocking agent use, tracheostomy and mechanical ventilation duration (dichotomized because of the non-linearity with the outcome).
