# Supplementary material for: High-speed trains versus air transport vectors for mass transfers of critically ill patients: The TRANSCOV cohort study
Source: PLoS One. 2026 Apr 28;21(4):e0348090. doi: 10.1371/journal.pone.0348090 (PMC13123964; doi:10.1371/journal.pone.0348090)
Supplement: S5 Fig — (DOCX) [file pone.0348090.s005.docx]

**S5 Fig. Associations between exposure and length of stay (log transformed) in subgroup univariate analyses.**


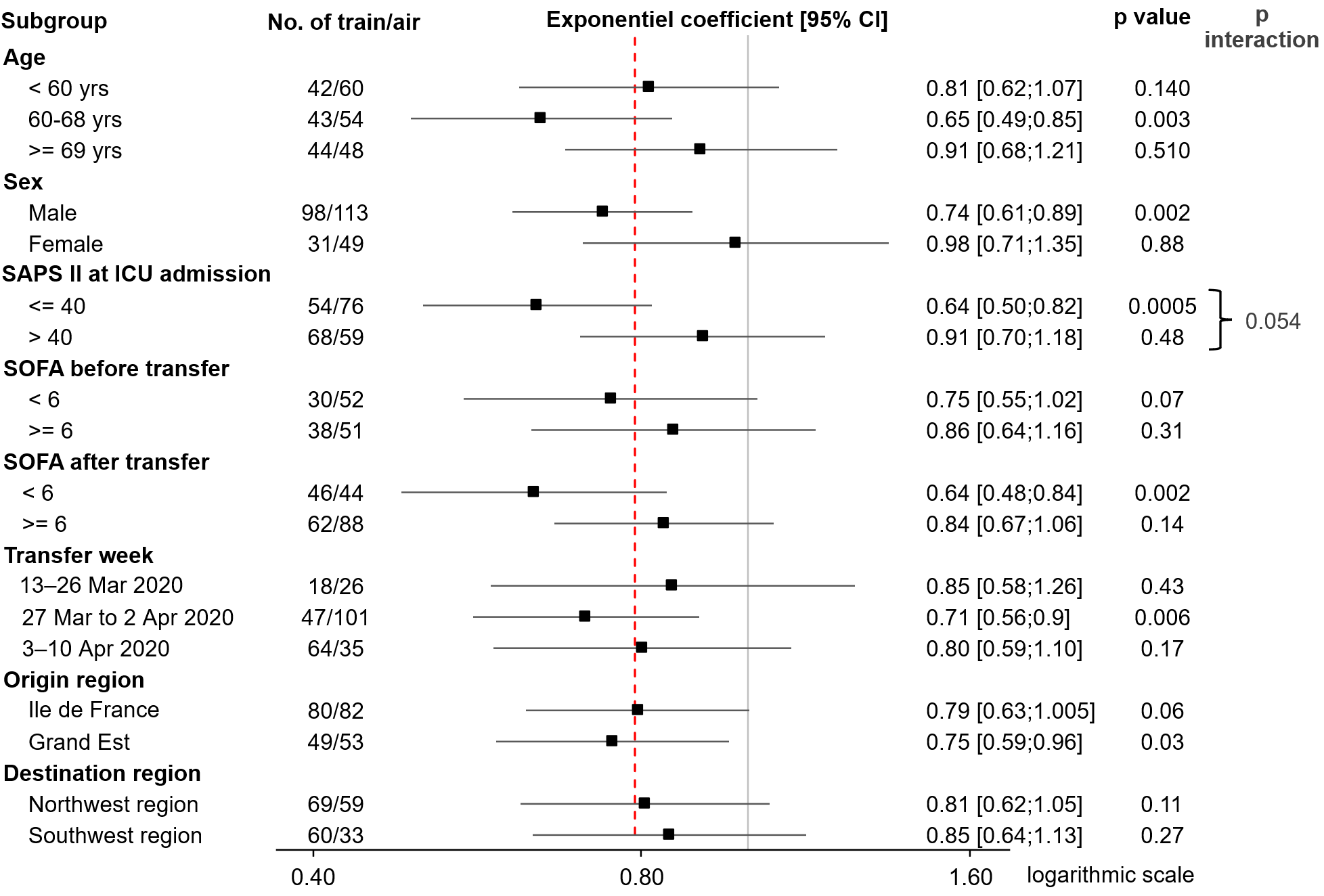


Results were from linear regression models using log-transformed length of stay as dependent variable. Therefore, the exponential coefficient can be interpreted as a multiplicative factor. For instance, the length of stay was 35% shorter [(1.00 - 0.65) x 100] for patients aged 60-68 years transferred by train compared to air. The dotted red line indicates the crude estimate for the total population (0.79 or 21% reduction in length of stay associated with train transfer). Although unadjusted analysis suggested the association was only present in men, similar associations were observed in both sexes when taking account age and initial SAPS II (not shown). ICU, intensive care unit; SAPS, Simplified Acute Physiology Score; SOFA, Sequential Organ Failure Assessment.
