# Supplementary material for: High-speed trains versus air transport vectors for mass transfers of critically ill patients: The TRANSCOV cohort study
Source: PLoS One. 2026 Apr 28;21(4):e0348090. doi: 10.1371/journal.pone.0348090 (PMC13123964; doi:10.1371/journal.pone.0348090)
Supplement: Attachment — Submitted filename: Responses to reviewers.docx [file pone.0348090.s006.docx]

## Plos ONE editor comments and questions

| 1.Please ensure that your manuscript meets PLOS ONE's style requirements, including those for file naming. The PLOS ONE style templates can be found at  <https://journals.plos.org/plosone/s/file?id=wjVg/PLOSOne_formatting_sample_main_body.pdf> and  [https://journals.plos.org/plosone/s/file?id=ba62/PLOSOne_formatting_sample_title_authors_affiliations.pdf](https://track.editorialmanager.com/CL0/https:%2F%2Fjournals.plos.org%2Fplosone%2Fs%2Ffile%3Fid=ba62%2FPLOSOne_formatting_sample_title_authors_affiliations.pdf/1/010f019c409e8ec9-c41d609d-56c4-4ec6-ba29-bccf8b7dce0b-000000/AbSK2GCbCHYb3ImUwmn8fpnbRq-26J-Tam0n3_RjVYw=249) |  |
| --- | --- |
| 2. Please provide additional details regarding participant consent. In the ethics statement in the Methods and online submission information, please ensure that you have specified what type you obtained (for instance, written or verbal, and if verbal, how it was documented and witnessed). If your study included minors, state whether you obtained consent from parents or guardians. If the need for consent was waived by the ethics committee, please include this information.  Once you have amended this/these statement(s) in the Methods section of the manuscript, please add the same text to the “Ethics Statement” field of the submission form (via “Edit Submission”).  For additional information about PLOS ONE ethical requirements for human subjects research, please refer to [http://journals.plos.org/plosone/s/submission-guidelines#loc-human-subjects-research](http://track.editorialmanager.com/CL0/http:%2F%2Fjournals.plos.org%2Fplosone%2Fs%2Fsubmission-guidelines%23loc-human-subjects-research/1/010f019c409e8ec9-c41d609d-56c4-4ec6-ba29-bccf8b7dce0b-000000/tDAbstKCGWBQ_WZDEGWy6BrZccHVwQJvLmTUXcobLag=249) | Indeed, the need for written or oral consent was waived by the ethics committee. We have modified the text in the paragraph “Ethics” to explain this more clearly. |
| 3. Please note that funding information should not appear in any section or other areas of your manuscript. We will only publish funding information present in the Funding Statement section of the online submission form. Please remove any funding-related text from the manuscript. | We have removed funding related text from the manuscript. Information regarding funding is now exclusively provided in the online submission form. |
| 4. In the online submission form you indicate that your data is not available for proprietary reasons and have provided a contact point for accessing this data. Please note that your current contact point is a co-author on this manuscript. According to our Data Policy, the contact point must not be an author on the manuscript and must be an institutional contact, ideally not an individual. Please revise your data statement to a non-author institutional point of contact, such as a data access or ethics committee, and send this to us via return email. Please also include contact information for the third party organization, and please include the full citation of where the data can be found. | Apologies for this mistake. The contact point is indeed the Data Protection Officer of our institution whose generic address is : [dpo@ehesp.fr](mailto:dpo@ehesp.fr). We have modified the data statement in the online submission form. |
| 5. One of the noted authors is a group or consortium “TRANSCOV Investigators”. In addition to naming the author group, please list the individual authors and affiliations within this group in the acknowledgments section of your manuscript. Please also indicate clearly a lead author for this group along with a contact email address. | Sorry, we initially provided the list of TRANSCOV investigators as a supplementary file “S2-Appendix.xlsx”. This appendix has been suppressed, and the list of the investigators is now included in the acknowledgments section of the manuscript. |
| 6. We note that there is identifying data in the Supporting Information file  “S2-Appendix.xlsx”. Due to the inclusion of these potentially identifying data, we have removed this file from your file inventory. Prior to sharing human research participant data, authors should consult with an ethics committee to ensure data are shared in accordance with participant consent and all applicable local laws.  Data sharing should never compromise participant privacy. It is therefore not appropriate to publicly share personally identifiable data on human research participants. The following are examples of data that should not be shared:  -Name, initials, physical address  -Ages more specific than whole numbers  -Internet protocol (IP) address  -Specific dates (birth dates, death dates, examination dates, etc.)  -Contact information such as phone number or email address  -Location data  -ID numbers that seem specific (long numbers, include initials, titled “Hospital ID”) rather than random (small numbers in numerical order)  Data that are not directly identifying may also be inappropriate to share, as in combination they can become identifying. For example, data collected from a small group of participants, vulnerable populations, or private groups should not be shared if they involve indirect identifiers (such as sex, ethnicity, location, etc.) that may risk the identification of study participants.  Additional guidance on preparing raw data for publication can be found in our Data Policy (<https://journals.plos.org/plosone/s/data-availability#loc-human-research-participant-data-and-other-sensitive-data>) and in the following article: <http://www.bmj.com/content/340/bmj.c181.long>.  Please remove or anonymize all personal information (<specific identifying information in file to be removed>), ensure that the data shared are in accordance with participant consent, and re-upload a fully anonymized data set. Please note that spreadsheet columns with personal information must be removed and not hidden as all hidden columns will appear in the published file. | S2_Appendix was meant to provide the list of TRANSCOV investigators, not of patients participants to the study who are fully anonymised.  As explained in answer to question 5 above, the S2_appendix has been suppressed, and the list of investigators can now be found in the acknowledgements section. |
| 7. If the reviewer comments include a recommendation to cite specific previously published works, please review and evaluate these publications to determine whether they are relevant and should be cited. There is no requirement to cite these works unless the editor has indicated otherwise. | Not applicable |
| Additional Editor Comments: In addition to the reviewer's comments, I would like to highlight one editorial point for your revision. Given the context of mass transfers of critically ill patients during an infectious disease outbreak, the manuscript would benefit from a more explicit discussion of infection prevention (IPC) aspects. Specifically, please consider addressing issues such as transmission risk during transport, mitigation strategies implemented in each transport modality, and the implications of your findings for IPC planning in future large-scale transfers.  I look forward to receiving your revised submission. | Thank you for raising this point. Unfortunately, patient to staff contaminations were not investigated during our study. Also, we are not aware of studies which have looked specifically at this issue. However, according to the experience of actors involved in the organisation and realisation of transfers, there were no “noticeable evidence of contamination above expected levels”.  We have added a few sentences at the end of the discussion section (last paragraph before conclusion), to address this point. |

Reviewers' comments:

Reviewer's Responses to Questions

**Comments to the Author**
1. Is the manuscript technically sound, and do the data support the conclusions?
The manuscript must describe a technically sound piece of scientific research with data that supports the conclusions. Experiments must have been conducted rigorously, with appropriate controls, replication, and sample sizes. The conclusions must be drawn appropriately based on the data presented.

Reviewer #1: Partly

2. Has the statistical analysis been performed appropriately and rigorously?

Reviewer #1: No

3. Have the authors made all data underlying the findings in their manuscript fully available?
The [PLOS Data policy](http://track.editorialmanager.com/CL0/http:%2F%2Fwww.plosone.org%2Fstatic%2Fpolicies.action%23sharing/1/010f019c409e8ec9-c41d609d-56c4-4ec6-ba29-bccf8b7dce0b-000000/8VpAiLzxt0KiVMgruPgjVDujn2UPRvdNCuaEl-BObH8=249) requires authors to make all data underlying the findings described in their manuscript fully available without restriction, with rare exception (please refer to the Data Availability Statement in the manuscript PDF file). The data should be provided as part of the manuscript or its supporting information, or deposited to a public repository. For example, in addition to summary statistics, the data points behind means, medians and variance measures should be available. If there are restrictions on publicly sharing data—e.g. participant privacy or use of data from a third party—those must be specified.

Reviewer #1: No

4. Is the manuscript presented in an intelligible fashion and written in standard English?

PLOS ONE does not copyedit accepted manuscripts, so the language in submitted articles must be clear, correct, and unambiguous. Any typographical or grammatical errors should be corrected at revision, so please note any specific errors here.

Reviewer #1: Yes

Reviewer #1:

Thank you for the opportunity to review this interesting manuscript. I have two main reservations:

| 1. Length of stay in the destination ITU is used as an outcome measure to compare Train vs Air transport. However, the destinations between the two groups differ significantly (Table 2) and importantly. This is a key confounder and the most likely reason for the findings with regards to a shorter length of stay in patients transferred by train. Since they arrived in ICUs further away from the epicentre of the pandemic with likely more capacity and less requirement to discharge patients rapidly from ITU. The authors mention this but do not highlight it sufficiently (I disagree that "physical & care environments during transfer provide a more compelling explanation" for a median LOS of 6 days less in the train group). The manuscript should be adapted to make this key limitation more explicit. A geographical map summarising destinations for Train group vs Air group may be helpful to the reader. Average length of stay in ITU for the period in question at each destination Hospital would be very useful to assess the likely impact of destination ITU but appreciate this data may not be available. | Thanks for raising this important point. It is true that confounding could arise from the fact that train patients travelled on average further away from the epicentres of the pandemic. Unfortunately we do not have access to information indicating workload or LOS in destination ICUs during the study period, which would have been ideal to address this issue.  However, we have stratified LOS post transfer according to terciles of transfer distance, as displayed in the table below.    Overall there does not seem to be a clear association between transfer distance and LOS. Also, for any given tercile of distance, LOS is always shorter in the Train group. Adding transfer-distance as an independent co-variable does not modify the association between train and shorter LOS. Altogether, this does not support the hypothesis of a confounding effect of distance.  We have added two sentences at the end of the 3^rd^ paragraph of the results sections to summarise these findings and also added a corresponding sentence in the discussion to address this point.  You are right that we don’t have hard evidence that better care environment in trains is the reason for shorter LOS in destination ICU. We have removed the word “compelling” and reworded the corresponding sentence in a more neutral way.  As suggested, we have included maps to the supplementary material to summarise transfers destination according to vectors for patients involved in the analysis (Shown in supplement S1 Fig). |
| --- | --- |
| 2. 208 patients have been excluded from the initial 501 transfers. 171 due to unknown destination and 20 for unknown vectors. The authors also note "high proportion of missing data for some key parameters". The percentage of missing data for each parameter needs to be provided as a supplementary table - I am concerned that the SOFA score comparison presented (lines 207 to 210) may be misleading if a large volume of data points are missing. I am generally concerned with the large percentage of missing data and conclusions must be suitably cautious. The manuscript must make it explicit to the reader as to the uncertainty of any conclusions drawn from this data. | Fair point.  The main cause of concern is the high level of missingness for pre-transfer SOFA score (48% and 37% respectively in the train and air groups). Following your advice, we are attracting the reader’s attention to this problem in the results section (see 2^nd^ paragraph of the results section). We highlight this problem again more explicitly in the discussion (see 3^rd^ paragraph of the discussion).  Of note, there is no association between missingness of pre-transfer SOFA and length of stay in destination ICU    One other variable present high level of missingness:   - “lung damage >50% from baseline CT scan” (in table 1, 45% and 58% missing in the train and air groups respectively).   If you agree, we propose to suppress this parameter altogether, since there is sufficient other information to compare the two groups according to clinical status at first admission. (nb: we have also removed this parameter from tables in supplemental material).  Please note that in table 2, we have modified numbers missing for the indicators “Noradrenaline dose” and Neuromuscular blockage duration. The missing numbers previously reported included patients who were not prescribed these drugs.  In tables 1-3 we have left the numerators and denominators used for deriving percentages of categorical variables, as well as presented the number missing (in brackets) for continuous variables. So that missingness can be estimated readily by readers. As requested, you will find at the end of the present document tables presenting percentages of missing data for each indicator presented in tables 1-3 of the main article (now without “lung damage”). Missingness is less than 10% for all remaining parameters but two (post transfer SOFA score (17% & 18%, train and air groups respectively) and ICU door to door duration of transfer (12% & 15%)). As you suggested, these tables could be presented as supplemental material. We feel that this could be redundant with the information available in the main article, but we will follow your decision on this matter. |

## Other points for consideration

| Line 82 - please include a comparison of what was available on trains in terms of procedures and tests vs air. | We have added a sentence to the introduction to clarify that a mobile laboratory and an echocardiograph were available on every train convoy. This was also the case on aeroplanes, but not on helicopters. We are currently working on a specific analysis comparing medical evacuations by aeroplane and helicopter using the Transcov database. This analysis requires further development, particularly due to a group of 43 air transfers for which we have no information enabling us to determine the type of aircraft used (aeroplane or helicopter). However, on the basis on available information, we can estimate that helicopter was the most frequent air vector (70%) used during our study period. |
| --- | --- |
| Paragraph from Line 85 to 90 should be in the methods section. | It is true that this paragraph describes the process or method used to organise the transfer. However, in our view, and given the retrospective nature of our study, these elements relate more to the context of the event that our study sought to assess than to our methodological choices. We would therefore prefer to keep this paragraph in the introduction, if that is acceptable to you. |
| Line 89 "noradrenaline <1mg/h" - was this not mcg/kg/min? mg/h | Good point, but we are quoting published guidelines which were formulated in this way. |
| Line 110-111 helicopter vs fixed-wing are very different platforms. Even if data is incomplete would be useful to know the breakdown in the known data. | Agreed, we are actually preparing a specific paper comparing Air vectors (cf answer above). |
| Line 135 "Catecholamine requirement" is used throughout the manuscript, does this exclude vasopressin? - would vasopressor/inotrope be preferable terminology? | Regarding this specific item, research assistants were proposed the following options in the CRF: 1) Noradrenaline, 2) Adrenaline 3) Dobutamine.  Only one patient was prescribed Dobutamine, so we did not include this variable specifically. Since vasopressin was not asked in the CRF, we believe that the generic term Catecholamine is correct. Vasopressor would suit us as well. |
| Line 206-207 Only the air group is presented whereas there is also a deterioration in the train group. Why is this not included? I would also ask for with-in group statistical analysis i.e paO2/Fio2 ratio before vs after in the train group & PaO2/Fio2 ratio before vs after in the air group as an assessment of statistically significant deterioration or not. | We have added in the written part of the results section the evolution of the PaO2/FiO2 ratio for both groups, as well as the p values of the before and after comparison for each group. Both groups deteriorate, the deterioration is larger (and statistically significant) in the air group. |
| Line 284 "they had better nursing care" - this may be true but by what measure? At the very least this should be qualified with "may" - it should also be balanced with the more likely factor that they arrived in less pressured ICUs who were able to provide days of better ICU care thereby reducing the risk of nosocomial resp infections as an alternative (to my mind more likely) explanation for the findings. | Fair comment.  We have amended the wording accordingly and added a sentence reminding the difference in equipment between the helicopter and other vectors, which we believe is relevant to this line of argument. |
| Line 304-305 a cost analysis is performed in the conclusion. This should be moved to the discussion. Furthermore, I personally think this should be removed as a gross over-simplification of results based on generally poor data & extrapolating a cost saving is mis-leading. | You are right that this is very much a “back of the envelope” estimation. We had planned in the overall TRANSCOV project to carry out a proper economic evaluation. However, it proved (frustratingly) impossible to collect the relevant cost data.  We are happy with your suggestion of removing the corresponding sentences. |
| Is there any information on the land transfer from hospital to airfield and airfield to hospital as this may be significant in some regions. | Unfortunately, we do not have any ‘concrete data’ to answer this question. As explained in the manuscript, we estimated the duration of transport. The duration of the ‘land transfer’ can therefore also be estimated very roughly by subtracting the ‘door-to-door’ time from the ‘estimated duration of the transport route’. We are aware that this is a very rough estimate, particularly for train transfers, given that some convoys stopped at a station to disembark a few patients before continuing on to their final destination. |
| I thank the authors again for an interesting article with potential implications for any future pandemics/major incidents. | Thank you very much for the insightful review and your constructive comments. |

**Missingness in the TRANSCOV database**

- **Table M1 : Missingness for demographic and pre-transfer clinical data (presented in table 1 of the main article)**
- **Table M2 : Missingness for transfer characteristics, clinical status before and after transfer (presented in table 2 of the main article)**
- **Table M3 : Missingness for clinical events, treatments and outcomes at the destination ICU (presented in table 3 of the main article)**

**Table M1 : Missingness for demographic and pre-transfer clinical data (presented in table 1 of the main article)**

| **Patient characteristics** | **Train group**  **N = 130** | **Air group**  **N = 163** |
| --- | --- | --- |
| Age | 0 (0%) | 0 (0%) |
| Men | 0 (0%) | 0 (0%) |
| Weight | 6 (4.6%) | 11 (6.7%) |
| Risk factors |  |  |
| Current smoking | 9 (6.9%) | 9 (5.5%) |
| Chronic alcoholism | 11 (8.5%) | 11 (6.7%) |
| Comorbidities |  |  |
| Any comorbidity | 0 (0%) | 0 (0%) |
| No. of comorbidities | 3 (2.3%) | 6 (3.7%) |
| Diabetes | 1 (0.3%) | 0 (0%) |
| Hypertension | 1 (0.3%) | 0 (0%) |
| Cardiovascular disease^b^ | 2 (1.5%) | 3 (1.8%) |
| Asthma or COPD | 3 (2.3%) | 2 (1.2%) |
| Functional limitations (KNAUS score) | 13 (10%) | 13 (8%) |
| Clinical status at admission in the origin ICU |  |  |
| Disease duration before ICU admission | 7 (5.4%) | 3 (1.8%) |
| SAPS II | 8 (6.2%) | 27 (16.6%) |
| Intubated at admission | 0 (0%) | 1 (0.6%) |
| LOS at the origin ICU | 1 (0.8%) | 1 (0.6%) |
| Data are reported n missing (%). COPD, chronic obstructive pulmonary disease; CT, computed tomography; ICU, intensive care unit; LOS, length of stay; SAPS, Simplified Acute Physiology Score.  ^b^Cardiovascular disease included either ischaemic heart disease, heart failure or stroke. | | |

**Table M2 : Missingness for transfer characteristics, clinical status before and after transfer (presented in table 2 of the main article)**

| **Variables** | **Train group**  **N = 130** | **Air group**  **N = 163** |
| --- | --- | --- |
| Transfer characteristics |  |  |
| Period of transfer | 0 (0%) | 0 (0%) |
| Origin region | 0 (0%) | 0 (0%) |
| Destination region | 0 (0%) | 0 (0%) |
| Distance (km) | 0 (0%) | 0 (0%) |
| Duration (hours) |  |  |
| ICU door to door | 16 (12.3%) | 25 (15.3%) |
| In the main transport vector | 0 (0%) | 0 (0%) |
| Pre- and post-transfer clinical status |  |  |
| SOFA score |  |  |
| Pre-transfer | 62 (47.7%) | 60 (36.8%) |
| Post-transfer | 22 (16.9%) | 30 (18.4%) |
| PaO_2_/FiO_2_ ratio (mmHg) |  |  |
| Pre-transfer | 5 (3.8%) | 5 (3.1%) |
| Post-transfer | 2 (1.5%) | 0 (0%) |
| Arterial pH |  |  |
| Pre-transfer | 4 (3.1%) | 1 (0.6%) |
| Post-transfer | 1 (0.8%) | 0 (0%) |
| PaCO_2_ (mmHg) |  |  |
| Pre-transfer | 4 (3.1%) | 1 (0.6%) |
| Post-transfer | 1 (0.8%) | 0 (0%) |
| Serum bicarbonate (mEq/L) |  |  |
| Pre-transfer | 7 (5.4%) | 2 (1.2%) |
| Post-transfer | 3 (2.3%) | 1 (0.6%) |
| Catecholamine requirement |  |  |
| Pre-transfer | 1 (0.8%) | 1 (0.6%) |
| Post-transfer | 2 (1.5%) | 0 (0%) |
| Noradrenaline dose (mcg/kg/min) |  |  |
| Pre-transfer | 4 (3.1%) | 3 (1.8%) |
| Post-transfer | 4 (3.1%) | 8 (4.9%) |
| Post-transfer SAPS II | 11 (8.5%) | 10 (6.1%) |
| Ventilation at admission in the destination ICU | 0 (0%) | 0 (0%) |
| Data are reported as n missing (%). FiO2, fraction of inspired oxygen; ICU, intensive care unit; PaCO2, arterial carbon dioxide partial pressure; PaO2, arterial oxygen partial pressure; SAPS, Simplified Acute Physiology Score; SOFA, Sequential Organ Failure Assessment. | | |

**Table M3 : Missingness for clinical events, treatments and outcomes at the destination ICU (presented in table 3 of the main article)**

|  | **Train group**  **N = 130** | **Air group**  **N = 163** |
| --- | --- | --- |
| Clinical events |  |  |
| Any clinical event | 0 (0%) | 1 (0.6%) |
| Shock | 0 (0%) | 1 (0.6%) |
| Acute kidney injury | 0 (0%) | 0 (0%) |
| Nosocomial infection | 0 (0%) | 0 (0%) |
| Thromboembolic event | 0 (0%) | 2 (1.2%) |
| Encephalitis | 0 (0%) | 1 (0.6%) |
| Epilepsy | 0 (0%) | 0 (0%) |
| Delirium | 1 (0.8%) | 1 (0.6%) |
| Other psychological problems | 5 (3.8%) | 5 (3.1%) |
| Neuromyopathy | 0 (0%) | 3 (1.8%) |
| Treatments |  |  |
| Neuromuscular blocking agents | 2 (1.5%) | 0 (0%) |
| Neuromuscular blockage duration (days) | 10 (7.7%) | 14 (8.6%) |
| Prone position | 0 (0%) | 0 (0%) |
| Tracheostomy | 0 (0%) | 0 (0%) |
| ECMO | 0 (0%) | 0 (0%) |
| Mechanical ventilation duration (days) | 8 (6.2%) | 20 (12.3%) |
| Outcomes at the destination ICU |  |  |
| Length of stay (days) | 1 (0.8%) | 1 (0.6%) |
| Death in ICU | 0 (0%) | 0 (0%) |
| 28-day mortality | 0 (0%) | 0 (0%) |
| Data are reported as n missing (%). ECMO, extracorporeal membrane oxygenation; ICU, intensive care unit; SAPS, Simplified Acute Physiology Score. | | |
